# Lyophilized Polyvinylpyrrolidone Hydrogel for Culture of Human Oral Mucosa Stem Cells

**DOI:** 10.3390/ma14010227

**Published:** 2021-01-05

**Authors:** Carolina Oliver-Urrutia, Raúl Rosales Ibañez, Miriam V. Flores-Merino, Lucy Vojtova, Jakub Salplachta, Ladislav Čelko, Jozef Kaiser, Edgar B. Montufar

**Affiliations:** 1 Faculty of Chemistry, Autonomous University of the State of Mexico, Paseo Colon S/N, Toluca 50120, Mexico; mv.flores.merino@gmail.com; 2 Central European Institute of Technology, Brno University of Technology, Purkynova 123, 61200 Brno, Czech Republic; lucy.vojtova@ceitec.vutbr.cz (L.V.); ladislav.celko@ceitec.vutbr.cz (L.Č.); jozef.kaiser@ceitec.vutbr.cz (J.K.); eb.montufar@ceitec.vutbr.cz (E.B.M.); 3 Faculty of Higher Studies Iztacala, National Autonomous University of Mexico, Los Reyes Iztacala 1, Mexico City 54090, Mexico; dr.raul.rosales@gmail.com

**Keywords:** polyvinylpyrrolidone, hydrogel, lyophilisation, nano-computed tomography, porosity, oral mucosa, stem cell

## Abstract

This work shows the synthesis of a polyvinylpyrrolidone (PVP) hydrogel by heat-activated polymerization and explores the production of hydrogels with an open porous network by lyophilisation to allow the three-dimensional culture of human oral mucosa stem cells (hOMSCs). The swollen hydrogel showed a storage modulus similar to oral mucosa and elastic solid rheological behaviour without sol transition. A comprehensive characterization of porosity by scanning electron microscopy, mercury intrusion porosimetry and nano-computed tomography (with spatial resolution below 1 μm) showed that lyophilisation resulted in the heterogeneous incorporation of closed oval-like pores in the hydrogel with broad size distribution (5 to 180 μm, d_50_ = 65 μm). Human oral mucosa biopsies were used to isolate hOMSCs, expressing typical markers of mesenchymal stem cells in more than 95% of the cell population. Direct contact cytotoxicity assay demonstrated that PVP hydrogel have no negative effect on cell metabolic activity, allowing the culture of hOMSCs with normal fusiform morphology. Pore connectivity should be improved in future to allow cell growth in the bulk of the PVP hydrogel.

## 1. Introduction

Polyvinylpyrrolidone (PVP) is a synthetic and hydrosoluble polymer produced by free radical polymerization [1,2]. The hydrophilic and hydrophobic functional groups allow PVP solubility in water and in several organic solvents, such as methanol, ethanol, chloroform, or propanol [1,2,3]. PVP is chemically inert and suitable as a stable biomaterial in numerous medical applications [2,4]. For instance, in the pharmaceutical industry, it is used as a carrier of some hydrophilic and hydrophobic drugs [1,3]. Moreover, PVP can encapsulate DNA to protect it from intracellular degradation [5]. One advantage of PVP is that it can be processed to obtain hydrogels [4,6,7]. Hydrogels are three-dimensional (3D) polymeric networks that can mimic both the biological and the mechanical properties of extracellular matrix [7,8,9]. Therefore, hydrogels can act as a cell-supporting material for cell encapsulation and delivery [7,8]. Furthermore, the porous network in the hydrogels is controlled in a variety of pore sizes and shapes that allow the exchange of nutrients and signalling molecules [9,10]. An optimal combination of porosity, pore size, pore connectivity and pore spatial distribution is a critical requirement for proper cell seeding and further homogeneous cell growth in the bulk of the hydrogel [11]. It has been demonstrated that the space between hydrogel polymeric molecules is not suitable for cell migration, leading to heterogeneous distributions of cells [12]. Hence, bigger pores are required for homogeneous cell ingrowth [11,13]. There are several methods for the production of structures with an open porous network in the scale of few hundreds of micrometres, including phase separation [14], porogen leaching [15], gas foaming [16], freeze-thawing [17,18], lyophilisation [19], and cryogelation [20]. Among them, lyophilisation is a process where ice crystals are formed and then removed through sublimation under vacuum [12,21]. Since hydrogels contain more than 50% of water, the frozen water acts as a green and safety porogen for the formation of porous structures (i.e., scaffolds) [12,19,21].

One possible use of PVP scaffolds is in the regeneration of tooth supporting structures (i.e., cementum, periodontal ligament and bone). The scaffold can be used for the transport and delivery of stem cells that promote healing and repair of oral tissues damaged by inflammatory diseases, such as periodontitis, which worldwide is the leading cause of tooth loss. Although blends of PVP with other polymers revealed no toxic effects on fibroblast and endothelial cells [22,23], the encapsulation of stem cells in PVP hydrogels requires further investigation. A variety of post-natal tissues have reservoirs of stem cells that contribute to the maintenance, development, homeostasis, and tissue regeneration [24,25,26]. In past, the stem cell harvesting required invasive procedures that limited their clinical utility. This limitation was reduced when stem cells started to be isolated from orofacial tissues, which are much more accessible [24]. The first stem cells isolated from the oral cavity were the dental pulp stem cells (DPSCs) [25]. Nowadays, more oral sites with stem cell reservoirs have been discovered, leading to the isolation of periodontal ligament stem cells (PDLSCs), gingiva-derived mesenchymal stem cells (GMSCs), dental follicle progenitor cells (DFPCs), and stem cells from apical papilla (SCAP) [26,27,28]. All these cells have the potential to differentiate in osteogenic and chondrogenic lineages, representing a suitable source of stem cells for therapeutic applications [24,29]. Moreover, the mesenchymal stem cells (MSCs) of dental origin showed immunosuppressive properties and an anti-inflammatory function [30,31] that may aid in tissue repair related to oral inflammatory infections.

The aim of this work was to explore heat-activated polymerization process to synthesize a crosslinked PVP hydrogel with storage modulus similar to oral mucosa stroma. In addition, pores with size in the scale of tens of micrometres were incorporated by lyophilisation to allow the 3D culture of human oral mucosa stem cells (hOMSCs). As prove in this study, hOMSCs were easily isolated form the retromolar region and expressed MSCs markers in more than 95% of the cell population. The cytocompatibility of the PVP hydrogel was shown for the first time in direct contact with hOMSCs. As the cell response depends on the mechanical microenvironment and porosity, the stiffness of the hydrogel was determined by rheological test and the porosity generated by lyophilisation was studied by X-ray nano-computed tomography (nano-CT), acquiring information about pore size distribution and connectivity with a spatial resolution below 1 µm. The results are encouraging to continue exploring PVP as a promising alternative to natural (alginate, collagen, agarose, chitosan) and synthetic (poly(ethylene glycol)) hydrogels for the encapsulation and delivery of stem cells.

## 2. Materials and Methods

### 2.1. Synthesis of Poly (N-vinylpyrrolidone) Hydrogel

Polymeric hydrogel networks were obtained by a sequential method previously described [32,33]. First, 1-vinyl-2-pyrrolidone (NVP; Sigma-Aldrich, St. Louis, MS, USA) was solubilized in distilled water (70 wt.%). Then, di-ethylene glycol bis-allyl carbonate (DEGBAC; Sigma-Aldrich, St. Louis, MS, USA) and azobisisobutyronitrile (AIBN; Sigma-Aldrich, St. Louis, MS, USA) were added (0.4 wt.% in 1:1 ratio). Polymerization of the solution was performed at 50 ± 2 °C for 24 h in glass beakers, sealed with aluminium foil inside an oven (Blue-M, TPS, Riverside, MI, USA). Subsequently, the hydrogel was placed in an ethanol/water (70%/30% *v*/*v*) solution for 48 h in order to remove any unreacted reagents. Finally, PVP hydrogel network was embedded in a 0.1 M phosphate buffer solution (PBS; pH = 7.4, Sigma Aldrich, St. Louis, MS, USA) until swollen equilibrium was reached. Finally, samples were stored at 4 °C until characterization.

### 2.2. Infrared Spectroscopic Analysis

For Fourier-transform infrared spectroscopy (FTIR) analysis (Hyperion 3000/Vertex, Bruker, Billerica, MA, USA) the PVP hydrogel was dried in vacuum and grinded in a mortar and pestle. Transmittance was measured in the wavenumber range between 4000 and 800 cm^−1^ with 32 scans per sample.

### 2.3. Raman Spectroscopy Analysis

Similar to FTIR analysis, ground and dry hydrogel powder was analysed by Raman spectroscopy (Confocal Raman Imaging system WITec alpha 300R, WITec, Ulm, BW, Germany). Raman scattering was excited using 352 and 440 nm wavelengths lasers. The laser power of 5 mW together with a 40× objective lens were used.

### 2.4. Rheological Measurement

The mechanical properties (storage (G’) and loss (G”) modulus) of the swollen hydrogel were measured by oscillatory frequency sweep rheological test (ARES-G2 rheometer, New Castle, DE, USA). Water saturated hydrogel films with 5 mm of thickness were cut into disks (diameter of 22 mm) to match the diameter of the parallel-plates of the rheometer (gap width of 5 mm). Three independent samples were analysed in a frequency range from 0.1 to 100 rad/s at a constant temperature of 25 °C.

### 2.5. Fabrication and Characterization of Porous Samples

The porous samples for cell culture were fabricated by lyophilisation process (Martin Christ Epsilon 2-10D lyophilizer, Osterode am Harz, Germany) as previously described [19]. Swollen hydrogels were frozen in 24-well cell culture plates at −35 °C under 100 Pa for 15 h, afterwards, lyophilized at 25 °C under 1 Pa for 24 h.

The porosity and pore size distribution of lyophilized hydrogels were analysed by mercury intrusion porosimetry (MIP, Poremaster Quantachrome, Boynton Beach, FL, USA) in the range between 0.009 and 150 µm. Scanning electron microscopy (SEM, Lyra 3 Tescan, Brno, Czech Republic) was employed to examine the morphology of lyophilized hydrogels. Samples were fixed on metal stubs by employing double-sided electrical conductive adhesive tapes and were coated with a 20 nm layer of carbon. The external surface and the longitudinal cross-sections of the hydrogel were observed. The pore size was determined using Image J 1.52a software (National Institutes of Health, Bethesda, MD, USA). Values were determined from three independently prepared samples. Furthermore, the total porosity of the hydrogel was determined by the hydrodynamic method in water at a constant temperature. The PVP hydrogel was weighed in a dry, immersed and wet state (maximum swelling). The percentage of porosity was obtained by the following equation,

% Porosity = (Ww − Wd)/(Ww − Wi)(1)

where, Ww and Wd represent the wet and dry weight of the material, respectively. Whereas, Wi is the weight of maximum swelled hydrogels immersed.

The 3D structure of lyophilized hydrogels was analysed by X-ray nano-CT (RIGAKU Nano3DX, Shibuya, Tokyo, Japan). Employed Nano3DX device was equipped with a 3300 × 2500 pixel^2^ X-ray CCD camera and a Cu rotatory target, working at an accelerating voltage of 40 kV and a current of 30 mA. An optical head with 20× magnification was chosen to reach the field of view at 700 × 900 μm^2^. The sample to detector distance was set to minimum, i.e., 0 mm. Binning 2 × 2 was used. All determining the linear voxel size of the resulting data at 0.54 μm. The size of sample was restricted to 800 × 400 × 300 μm^3^ to achieve such resolution. A total of 800 projections were taken with an exposure time of 12 s. The contrast between the material and the background was further increased using custom-written phase-retrieval software based on Paganin phase-retrieval algorithm [34]. Subsequently, tomographic data was reconstructed using ASTRA Tomography Toolbox (CWI, Amsterdam, The Netherlands) [35]. Visualization of the samples and quantifications of structural parameters were performed using VG Studio MAX 3.3 software (Volume Graphics GmbH, Heidelberg, Germany). A cuboid region of interest that fitted a representative volume of the scaffold was manually created. The surface determination tool was used to estimate the sample porosity and the foam structure analysis module was used to determine volumetric pore size distribution.

### 2.6. Isolation of Primary Human Oral Mucosa Cells

Primary human oral mucosa specimens were obtained in accordance with the Ethical Committee of the Medical Sciences Research Center, Autonomous University of the State of Mexico (authorization CEI CICMED 2019/01). All human donors were informed about the procedure and provided consent for biopsy and cell harvest. Samples were obtained from healthy human oral mucosa (retromolar region and maxillary tuberosity) through oral surgeries for clinical reasons. The oral mucosa samples were placed in 3 mL of transport medium consisting of 100 μm/mL streptomycin, 100 IU/mL penicillin, and 10 μm/mL amphotericin B in PBS (all from Sigma-Aldrich, St. Louis, MS, USA). Oral mucosa cells were obtained by the explant technique. Briefly, the epithelium was removed with a scalpel and the remaining connective tissue was aseptically cut into pieces of 1 mm^3^. The fragments were plated in 25 cm^2^ culture dishes and cultivated for 5 min at 37 °C, with 5% CO_2_ and 85% humidity. After the time, Dulbecco’s Modified Eagle Medium (DMEM, Biowest, Nuaille, Pays de la Loire, France) supplemented with 10% fetal bovine serum (FBS, Sigma Aldrich, St. Louis, MS, USA), penicillin and streptomycin (100 mg/mL, both from Sigma-Aldrich, St. Louis, MS, USA) was added and the fragments cultured at 37 °C, 5% CO_2_ and 85% humidity. After 14 days of culture the isolated cells were expanded in a new culture plate. Briefly, upon reaching 80% confluence, the cells were digested with 0.05% trypsin containing 1 mM EDTA (Sigma-Aldrich, St. Louis, MS, USA), and passaged. Cells on the third passage were used for the biological assays presented in this study. The culture medium was replaced every three days.

### 2.7. Flow Cytometry Analysis of Isolated Human Oral Mucosa Cells

Mesenchymal stem cells derived from human oral mucosa were analysed for cell surface markers expression (Cluster differentiation; CD) using a flow cytometer (CytoFLEX LX, Beckman Coulter, Indianapolis, IN, USA). Briefly, after three passages the cells were trypsinized, collected, and washed twice with PBS. The cell density was adjusted to 1 × 10^5^ cells/mL of PBS and 100 μL of the suspension was transferred into a fresh Eppendorf tube. The cells were incubated in dark with CD90 (Thy-1/Thy-1.1; FITC conjugated), CD73 (ecto-5′-nucleotidase; PECY7 conjugated) and CD105 (Endoglin; VB421 conjugated) for 30 min at 4 °C. All the antibodies were purchased from Abcam plc, Cambridge, UK. The cells were washed twice with PBS. Labelled cells were measured by flow cytometer using CytExpert software (Beckman Coulter, Indianapolis, IN, USA).

### 2.8. Immunocytochemical Analysis

Oral mucosa cells were seeded at a density of 8 × 10^4^ cells/well in a 96-well cell culture plate. After 24 h of culture, the cells were fixed in 10% formalin (Sigma-Aldrich, St. Louis, MS, USA) for 20 min and permeabilized in 0.025% Triton X-100 (Sigma-Aldrich, St. Louis, MS, USA) for 20 min. Subsequently, inhibition in 1% bovine serum albumin (BSA, Sigma-Aldrich, St. Louis, MS, USA) for 30 min was done. Cells were labelled independently with anti-human monoclonal primary antibodies CD29, CD90, and Stro-1 (1:100, Santa Cruz Biotechnology, Dallas, TX, USA). As a secondary antibody, Alexa Fluor 488 (Anti-Rabbit IgG polyclonal, Invitrogen, Waltham, MA, USA) was used. The primary antibodies and the secondary antibodies were incubated for 2 h under dark at room temperature, performing three rinses with PBS between the two steps. Fluorescent images were captured with a fluorescence microscope (Zeiss HXP 120 C, Zeiss, Oberkochen, Germany) using a proper set of filters and ZEN LITE 201 Software (Zeiss, Oberkochen, Germany) was used for image acquisition.

### 2.9. Cytotoxicity Assay

The hOMSCs (8 × 10^4^ cells/well) were seeded in direct contact with fully-swollen PVP hydrogels (4 mm in diameter by 6 mm in height) in a 96-well cell culture plate. The cells and the PVP hydrogel were incubated together in 300 µl of supplemented DMEM for 3, 7 and 10 days at 37 °C, under 5% CO_2_ and 85% humidity. At such time points, the cells were washed twice with 100 µL of PBS. Then, 300 µL of phenol red-free medium (Biowest, Nuaille, Pays de la Loire, France) containing 10% AlamarBlue™ reagent (Thermo Fisher Scientific Inc., Waltham, MA, USA) were added to each well and further incubated overnight, protected from light. Afterward, 150 µL of the reacted mixture per sample were transferred to a new 96-well plate and fluorescence (excitation at 560 nm, emission at 590 nm) was measured using a microplate reader (Elx808, Biotek, Winooski, VT, USA). Cells cultured without hydrogel under the same conditions were used as control.

### 2.10. Statistical Analysis

Statistical analysis was performed by two-way analyses of variance (ANOVA) using GraphPad Prism 4 software (GraphPad, San Diego, CA, USA). Differences were considered statistically significant when *p* < 0.05. The results are shown as mean ± standard deviation (SD), where *n* represents the number of experimental samples.

## 3. Results

### 3.1. Chemical Composition of PVP Hydrogel

**Figure 1:**
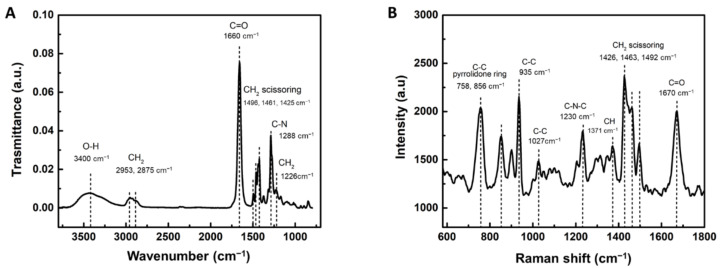
Chemical analysis of PVP hydrogel by (**A**) FTIR and (**B**) Raman spectroscopies.

The FTIR spectrum of the PVP hydrogel is shown in Figure 1A. The vibration of the methylene group (CH_2_) is observed at 2953 and 2875 cm^−1^. A strong peak corresponding to carbonyl stretching (C=O) is found at 1660 cm^−1^. Medium bands are shown at 1496, 1461, and 1425 cm^−1^ corresponding to CH_2_ scissoring vibrations. C-N vibration is presented at 1288 cm^−1^. The peak at 1226 cm^−1^ is due to the CH_2_ vibration.

Raman spectrum of PVP hydrogel is shown in Figure 1B. The intense band of carbonyl group vibration (C=O) is observed at 1670 cm^−1^. The peaks at 1492, 1463, 1426 cm^−1^ are assigned to CH_2_ scissoring vibration. While the peak at 1371 cm^−1^ is assigned to CH group vibration. The C–N–C and C–C bands are presented at 1230 cm^−1^ and 1027 cm^−1^, respectively. The peak at 935 cm^−1^ is ascribed to C-C ring breathing. Also, 856 and 758 cm^−1^ peaks corresponded to C-C vibrations of pyrrolidone ring.

### 3.2. Oscillatory Frequency Sweep

**Figure 2:**
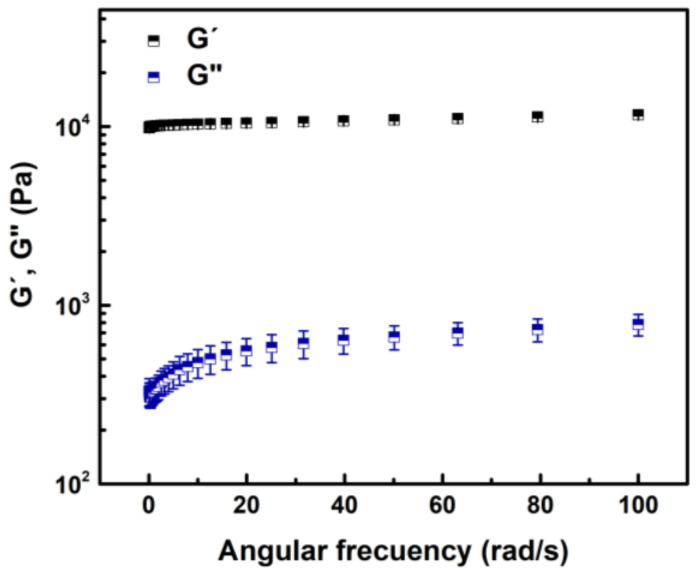
The relationship of storage (**G’**) and loss (**G”**) modulus with angular frequency of oscillation for PVP hydrogel at 25 °C. Results are expressed as the mean and standard deviation of the three independent measurements.

Figure 2 shows that the loss modulus (G”) increases while the storage modulus (G’) was constant with the frequency of oscillation, suggesting a moderated time-dependent rheological behaviour. Storage modulus (11.04 ± 0.36 kPa) was always 1 to 2 orders of magnitude greater than loss modulus (0.73 ± 0.04 kPa). Therefore, according to rheological principles [36,37,38], PVP is in gel form (solid-like), it does not show sol transition and is mechanically strong.

### 3.3. Porosity Analyses of Lyophilized Hydrogel

**Figure 3:**
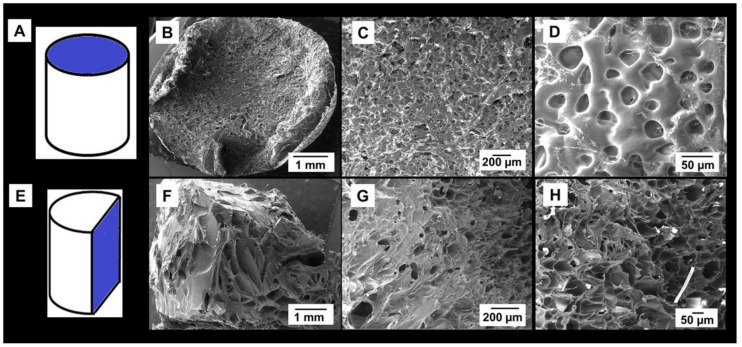
Representative secondary electron—SEM images of the microstructure of lyophilized PVP hydrogel: (**A**–**D**) top surface and (**E**–**H**) longitudinal cross-section.

Figure 3 shows the porous structure of the lyophilized hydrogel. The surface of the sample showed an equiaxed open cell pore morphology with an average pore size of around 40 μm and low dispersion (±7 μm) (Figure 3A–D). In contrast, the interior of the sample presented pores with heterogeneous size and complex morphology (Figure 3E–H).

**Figure 4:**
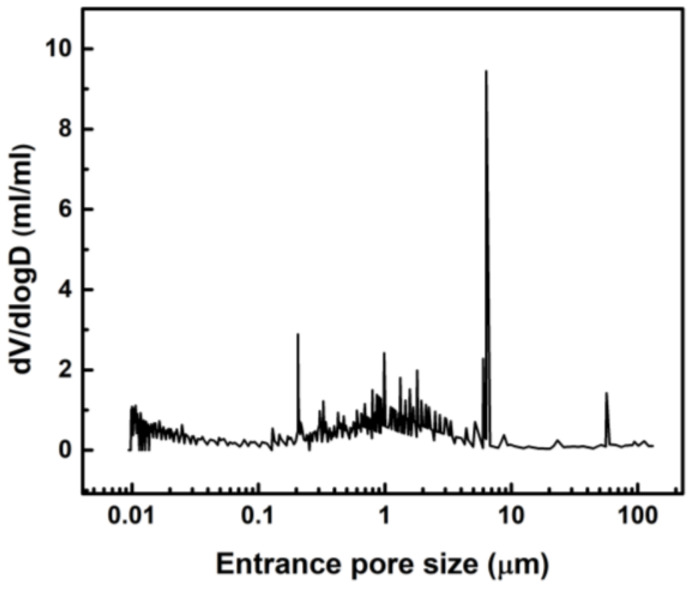
Entrance pore size distribution of lyophilized PVP hydrogel obtained by MIP.

The MIP analysis revealed a multimodal pore size distribution with well-defined peaks at 0.2, 6.3, and 60 μm; and a broad hump in the range from 0.1 to 6 μm (Figure 4). The larger pores detected by MIP corresponded to the open pores observed by SEM at the sample surface. In fact, the pore size is in good correlation between the two techniques, differences attributed to the principle of pore size measurement of MIP. MIP also showed smaller pores inside the samples (below 10 μm) and confirms the heterogeneity of their size. The open porosity determined by MIP was 65%, while the total porosity, determined by the hydrodynamic method, was 86%. The difference attributed to the range of analysis of MIP (from 0.009 to 150 µm in this study) and the presence of closed pores.

**Figure 5:**
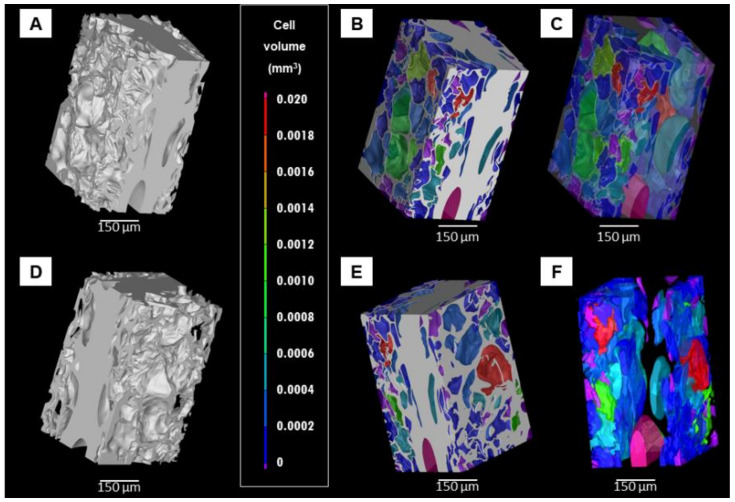
Three-dimensional (3D) virtual reconstructions of a lyophilized PVP hydrogel obtained by nano-CT. (**A**,**D**) Two different views of the material. (**B**,**C**,**E**,**F**) Pore volume distribution in colour-scale, images C and F are the same than images B and E, respectively, but the material was defined transparent for better observation of the pore morphology and distribution.

**Figure 6:**
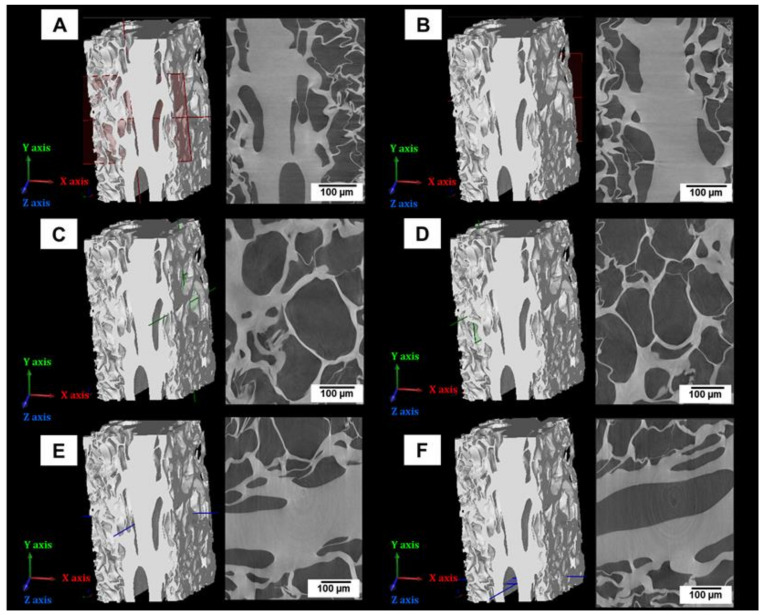
Representative images of orthogonal planes of the lyophilized PVP hydrogel obtained by nano-CT. The images (right side) were acquired in orthogonal planes of the virtual reconstruction (left side): (**A**,**B**) XY-plain in red colour, (**C**,**D**) YZ plain in green colour and (**E**,**F**) XZ-plain in blue colour.

The porosity determined by nano-CT was 43%. Figure 5 and Figure 6 show the 3D virtual reconstruction of the lyophilized hydrogel (the material is presented in grey colour). The volumetric size distribution of the pores is visualized by a colour scale in the Figure 5B–F. Moreover, in order to have a better perspective of the pore morphology and distribution, the material was set transparent in the visualizations shown in Figure 5C,F. The presence of a large number of pores implies that they are not connected, at least by apertures bigger than 1.5 μm (size of the smallest detectable feature). If the pores were open they would appear in one single colour self-connected and running continuously along the sample. Furthermore, the hydrogel presented heterogeneity in the spatial distribution of pores. The smooth surfaces of the material at the edges of the reconstruction appeared due to the cutting planes virtually applied to define the region of interest.

**Figure 7:**
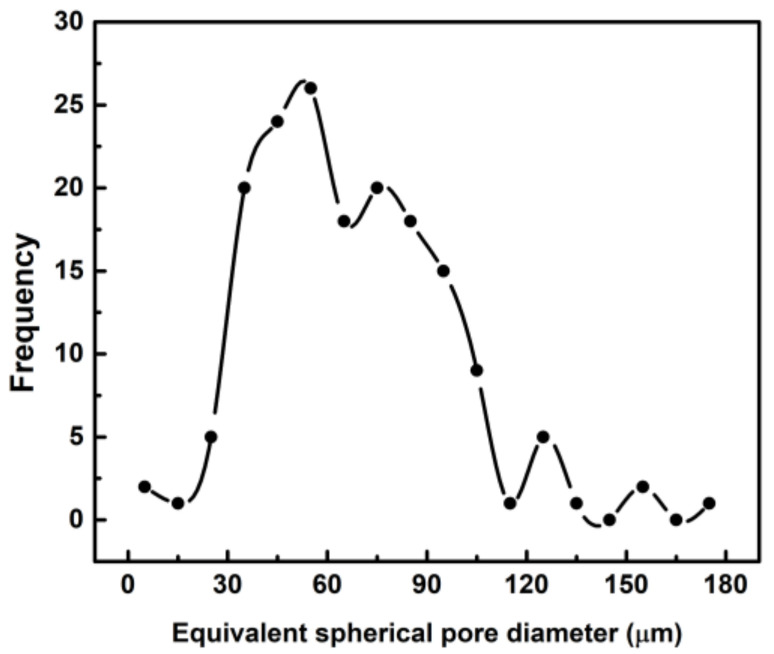
Histogram of the pore size distribution of the lyophilized PVP hydrogel determined by nano-CT analysis. Pore size is indicated as the equivalent spherical diameter of the pores observed in Figure 5.

Orthogonal cross-sections of the sample show that in general the pores had an oval-like morphology (Figure 6). The pores had a broad size distribution (Figure 7), the smallest pores had an equivalent spherical diameter of around 5 μm, while the largest pores presented an equivalent spherical diameter of around 180 μm. The median diameter of the pores (d_50_) was 65 μm, whereas the average diameter was 67 ± 29 μm.

### 3.4. Primary Human Oral Mucosa Stem Cells

**Figure 8:**
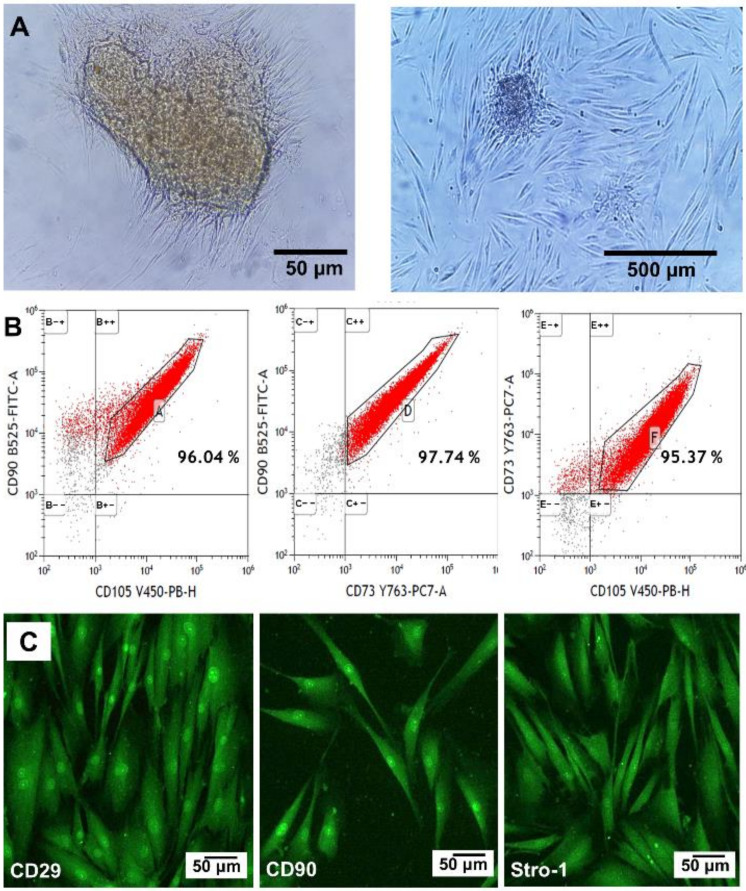
Characterization of human oral mucosa stem cells (hOMSCs). (**A**) Optical microscope images of the cells isolated from tissue explant after 14 days of culture. (**B**) Flow cytometry profiles and percentage of cells expressing CD90, CD105 and CD73, among the population of cells. (**C**) Fluorescence microscopy of selected mesenchymal stem cell markers (CD29, CD90 and Stro-1).

Isolated human oral mucosa cells grew in clusters and exhibited fusiform fibroblast-like morphology after 14 days of culture (Figure 8A). The cells showed 96.04% positivity towards CD90 and CD105, 97.74% positivity towards CD90 and CD73, and 95.37% positivity towards CD73 and CD105 (Figure 8B). Moreover, the immunostaining showed the presence of CD29, CD90, and Stro-1 proteins on the cell membrane (Figure 8C). All of them membrane markers of MSCs according to the International Society for Cellular Therapy [27,28,39].

### 3.5. Hydrogel Cytotoxicity

**Figure 9:**
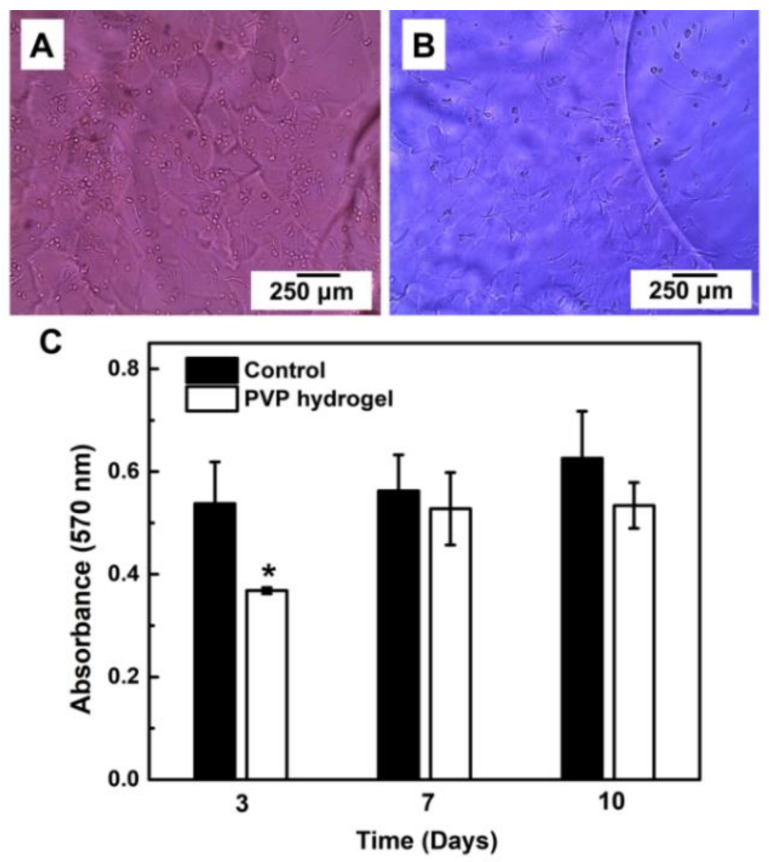
Hydrogel’s cytotoxicity against human oral mucosa stem cells (hOMSCs). (**A**,**B**) Optical microscope images of the cells in direct contact with PVP hydrogel at day 3 and 7, respectively. (**C**) Cell metabolic activity, results expressed as mean ± SD, (*) represents significant difference (*p* < 0.05) between groups and throughout days (n = 3; two-way ANOVA, Bonferroni).

A fusiform shape of the cells in direct contact with the hydrogel was observed on day 3 and 7 (Figure 9A,B). The cell metabolic activity in the control group (without hydrogel) did not have a statistically significant variation over the time (Figure 9C). The hydrogel generated a reduction (*p* < 0.05) of the cell metabolic activity after 3 days, showing 68.4% of activity respect to the control group (Figure 9C). Therefore, according with the ISO standard 10993-5, the hydrogel is 2% below the threshold to be consider cytocompatible. After this lag phase, the cell metabolic activity recovered a value close to the control group (93.8%), with no statistically significant differences in comparison to the control group at day 7 and 10 (Figure 9C). Therefore, the hydrogel did not exhibit a sustained cytotoxic effect on the primary hOMSCs.

## 4. Discussion

Heat-activated polymerization was used to fabricate a PVP hydrogel. This method is widely used in polymer network synthesis [3,6,12]. It is driven by the homolytic thermal dissociation of the initiator to create free radicals, allowing the formation of covalent bonds between the polymer chains. The chemical composition of PVP determined by FTIR and Raman spectroscopies was in agreement with previous reports where it was polymerized by different routes [3,4,18,40]. Furthermore, heat-activated polymerization produced a stable PVP hydrogel with clear elastic solid behaviour able to recover stored energy. In general, similar solid behaviour is reported for a wide range of PVP formulations and oscillation frequencies [4,37,38]. In particular, the PVP hydrogel developed in this work presented a storage modulus between keratinized gingiva (20 kPa) and tongue and soft palate (2.5 kPa) [41,42], the main tissues forming the oral mucosa, which is the target of this study to culture and retain undifferentiated the hOMSCs. Moreover, the processing conditions, such as cross-linker content, monomer concentration, amount of initiator, or increasing number of ionic groups, can be adjusted to approach even more the storage modulus to the specific site of the oral mucosa of interest. This is important since the mechanical properties are known to greatly influence cell fate. For example, Cameron et al. showed that increasing the loss moduli of polyacrylamide gels from 1 Pa to 130 Pa increases human MSCs spread area and proliferation [43]. Other works demonstrated different cell growth rates in response to stiffness. In general, stiffer hydrogels promote stem cell proliferation compared to softer gels [44].

In addition to the mechanical properties, porosity regulates cell attachment, cell interactions, cell migration, and differentiation [11,29,45]. Moreover, porosity affects the mechanical behaviour of the hydrogels. It was demonstrated that Young’s modulus of hydrogels with low porosity (60%) and small pore size (40 µm) was more than three times higher than Young’s modulus of hydrogels with 89% of porosity and higher pore size (125 µm) [46]. In this study, lyophilisation allowed the production of porous structures. During freezing, the water in the hydrogel form ice crystals that became pores after drying [12,21]. Under the optimal conditions, the drying of the sample by sublimation does not change the morphology of the ice crystals [12,21]. Therefore, freezing is the most important step to control the size and shape of pores. In this study, the freeze-drying conditions were selected according to the optimal parameters set to obtain collagen scaffolds [19]. It was found that the pore size distribution for the PVP (5 to 180 μm) differed from the pore size observed before in collagen (70 to 110 μm), indicating that the freeze-drying conditions cannot be generalized. There is not information about the optimum pore size to restore all the tooth supporting structures. Moreover, the optimum pore size may vary between the different oral tissues, but the lyophilized PVP may allow the regeneration of skin that grows in pores between 20 and 125 μm [47], being the tissue that most resembles the oral mucosa. It was also observed that scaffolds with a diverse pore size (50 to 100 µm) promoted stem cell growth with a significantly lower level of differentiation compared with scaffolds with a narrow pores size distribution, either with bigger (125 µm) or smaller (40 µm) pores [46]. Furthermore, unequally pore size distribution may provide an environment for MSC adhesion and invasion promoting viability and metabolism [45].

A comprehensive characterization of porosity was performed in this study. In addition to MIP and SEM, nano-CT was used to characterize the pore size distribution and morphology with submicron spatial resolution in 3D, providing additional information not obtained with SEM and MIP. Specifically, the achieved linear voxel size of the nano-CT data was 0.54 μm, when normally the CT data have voxel size values around tens of μm. The improved resolution was a clear advantage of the nano-CT analysis, since the size of the smallest feature detectable in the virtual reconstruction is around 1.5 μm. The most frequent pores detected by nano-CT had an equivalent sphere diameter of 65 μm (Figure 7), a value in very good agreement with the highest entrance pore size detected by MIP (60 μm, Figure 4). Moreover, these values are close to the pore size measured by SEM image analysis at the surface of the material (40 μm). Unlike MIP and SEM, nano-CT uncovered that lyophilisation produced a heterogeneous spatial distribution of pores, with some fractions of the sample free of pores. This may be due to the gradients of temperature during freezing and the physical interactions between the PVP molecules [19]. Furthermore, nano-CT detected only closed pores, meaning that the open pores detected by MIP at the sample surface corresponded to blind pores. The presence of closed pores explains the difference between the total porosity measured by the hydrodynamic method (86%) and the porosity detected by MIP (65%). Or in other words, the material has around 20% of closed pores. There is also a significant difference between the porosity determined by MIP (65%) and nano-CT (43%). This difference is attributed to submicrometric channels linking the micrometric pores, likely between the molecules of PVP. It should bear in mind that the quantity of submicrometric channels does not correspond to the difference between the porosity detected by MIP and nano-CT, due to the bottleneck effect related to the MIP principle of measurement [48]. Though the pores are connected by submicrometric channels, the size of such channels is not suitable to allow cell migration and colonization of the scaffold. Therefore, other lyophilisation conditions (speed, temperature, pressure) that result in open pores must be required.

This work demonstrates a simple method for the isolation of hOMSCs, expressing the membrane markers that characterize the MSCs. Furthermore, the results uncover that heat-activated polymerization produced a cytocompatible PVP hydrogel, which only produced a transient reduction of cell metabolic activity on day 3, recovering normal activity after 7 and 10 days of culture. The short-term reduction of the cell metabolic activity may be explained by the mechanical adaptation of the stem cells to the hydrogel [49]. In fact, the stiffness of polystyrene is eight orders of magnitude higher than the one of the PVP hydrogel. The cytocompatibility of PVP was in agreement with previous results, where PVP networks did not show toxic effects on mouse subconjunctival tissue cells, human dermal fibroblasts, and monocytes [22,23,32,38]. Similarly, poly (ε-caprolactone) and PVP networks did not show cytotoxicity, allowing the proliferation of MSCs [50]. The long term purpose of culturing hOMSCs in the PVP hydrogel is to use it as cell delivery system to restore periodontally damaged tooth supporting structures. Oral mucosa grafts have been successfully used in urethral and spinal cord reconstruction, as well as repair of calvarial and mandibular defects [51,52,53,54]. Oral mucosa is a less invasive source for MSCs harvesting than bone marrow or even tooth pulp. In general, oral stem cells have higher proliferation rate than bone marrow MSCs, allowing faster expansion [27]. These cells can differentiate into osteoblasts, adipocytes, chondrocytes and astrocytes [28,54], and have immunomodulatory and anti-inflammatory properties [27,30,54]. Further experiments will be performed to improve pore connectivity in PVP, allowing the evaluation of cell proliferation, migration, phenotype, and differentiation in 3D.

## 5. Conclusions

Heat-activated polymerization produced a PVP hydrogel with clear elastic solid behaviour in a wide range of oscillation frequencies and storage modulus similar to oral mucosa stroma. The rheological behaviour of the hydrogel was independent of time, did not show sol transition, and PVP chemical composition was in agreement with previous reports. The porosity analysis showed that lyophilisation resulted in the heterogeneous incorporation of 43% of closed pores in the hydrogel with a median equivalent spherical diameter of 65 μm. Therefore, the freeze-drying conditions should be optimized to promote pore connectivity through big apertures that allow cell colonization. Cytotoxicity assay demonstrated the ability of PVP hydrogel to support hOMSCs culture for up to 10 days, suggesting that the lyophilized hydrogel may provide a favourable environment for cells to adhere and proliferate. The optimal parameters for the 3D culture of the hOMSCs have to be elucidated in future.

## Data Availability

The data that support the findings of this study are contained within the article.
